# Beyond Borders: Investigating the Mysteries of Cacipacoré, a Lesser-Studied Arbovirus in Brazil

**DOI:** 10.3390/v16030336

**Published:** 2024-02-22

**Authors:** Marielena V. Saivish, Maurício L. Nogueira, Shannan L. Rossi, Nikos Vasilakis

**Affiliations:** 1 Laboratórios de Pesquisas em Virologia, Departamento de Doenças Dermatológicas, Infecciosas e Parasitárias, Faculdade de Medicina de São José do Rio Preto, Sao Jose do Rio Preto 15090-000, SP, Brazil; marielenasaivish@gmail.com (M.V.S.); mauricio.nogueira@edu.famerp.br (M.L.N.); 2 Brazilian Biosciences National Laboratory, Centro Nacional de Pesquisa em Energia e Materiais (CNPEM), Campinas 13083-100, SP, Brazil; 3 Department of Pathology, University of Texas Medical Branch, Galveston, TX 77555-0609, USA; slrossi@utmb.edu; 4 Center for Vector-Borne and Zoonotic Diseases, University of Texas Medical Branch, Galveston, TX 77555-0609, USA; 5 Institute for Human Infection and Immunity, University of Texas Medical Branch, Galveston, TX 77555-0610, USA

**Keywords:** *Orthoflavivirus*, transmission cycles, epidemiology, pathogenesis, clinical manifestations

## Abstract

Cacipacoré virus (CPCV) was discovered in 1977 deep in the Amazon rainforest from the blood of a black-faced ant thrush (*Formicarius analis*). As a member of the family Flaviviridae and genus orthoflavivirus, CPCV’s intricate ecological association with vectors and hosts raises profound questions. CPCV’s transmission cycle may involve birds, rodents, equids, bovines, marsupials, non-human primates, and bats as potential vertebrate hosts, whereas *Culex* and *Aedes* spp. mosquitoes have been implicated as potential vectors of transmission. The virus’ isolation across diverse biomes, including urban settings, suggests its adaptability, as well as presents challenges for its accurate diagnosis, and thus its impact on veterinary and human health. With no specific treatment or vaccine, its prevention hinges on traditional arbovirus control measures. Here, we provide an overview of its ecology, transmission cycles, epidemiology, pathogenesis, and prevention, aiming at improving our ability to better understand this neglected arbovirus.

## 1. Introduction

Orthoflaviviruses are members of a distinct group of arthropod-borne viruses (arboviruses), transmitted primarily through the bites of mosquitoes and ticks [1], whose impact on human health has global implications [2]. While epidemic orthoflaviviral diseases (e.g., dengue and Zika) capture immediate attention, their endemic counterparts (e.g., Ilheus, Rocio, and others) often do not receive the same attention, despite their profound and lasting impact on exposed populations [3,4,5,6,7,8]. This paradox is particularly pertinent in Brazil, a nation with optimal ecological conditions supporting the year-long breeding of mosquito vectors, coupled with socio-economic factors that facilitate mosquito proliferation and amplify human exposure to arthropod bites [9,10,11,12,13]. Notably, urban and peri-urban areas face the continuous threat of orthoflavivirus infections, mostly the dengue (DENV) and Zika (ZIKV) viruses, which places an immense burden on public healthcare systems, as well as socioeconomic disruptions across the tropics [14,15,16,17].

Brazil is a hotspot for arboviral diseases, mainly due to its constellation of biodiverse ecotypes, which include: (a) the Amazon basin, encompassing the largest rainforest in the world [18,19,20,21,22], (b) the Atlantic forest, with the largest biodiversity and extending as far south and inland as Argentina and Paraguay [23,24,25,26,27], (c) the Pantanal, considered to be the largest wetland globally [6,28,29,30,31,32,33,34], and (d) the Cerrado, a vast region of tropical savanna in eastern Brazil [35,36,37,38].

In this review, we summarize our current understanding of the Cacipacoré virus’ (CPCV) host range, transmission cycles, and epidemiology, as well as its pathogenesis and the clinical outcomes of infection against the backdrop of Brazil’s complex arboviral landscape. As the country grapples with the endemicity of DENVs [39,40], ZIKV [15,41,42], chikungunya virus (CHIKV) [43,44,45,46], and the perennial risk of yellow fever epizootics [26,47,48], the potential of emerging flaviviruses necessitates closer scrutiny. With lessons learned from the ZIKV introduction in the Western Hemisphere in 2013 and the subsequent epidemic [14,49], understanding the biology, transmission dynamics, and epidemiology of neglected orthoflaviviruses becomes imperative for public health preparedness and response. Through this review, we aim to underscore the importance of proactive research in mitigating the potential impact of CPCV on public and veterinary health.

## 2. History, Taxonomy, and Classification

CPCV derived its name from the Cacipacoré River in Pará State, Brazil, where it was initially isolated from the whole blood of an adult male *Formicarius analis*, commonly referred to as the ‘black-faced ant thrush’. This isolation took place near Cachoeira Porteira, in the municipality of Oriximiná, Pará, in July 1977, through the collaborative efforts of researchers from the Instituto Evandro Chagas (led by Dr. Amélia Travassos da Rosa) and the Pan American Health Organization (Dr. Francisco de Paula Pinheiro) [50,51].

Currently, the International Committee on Taxonomy of Viruses (ICTV) recognizes four genera within the *Flaviviridae* family: *Hepacivirus*, *Pegivirus*, *Pestivirus*, and *Orthoflavivirus* (previously referred to as *flavivirus*) [2]. The *Orthoflavivirus* genus comprises over 70 virus species, with mosquitoes and ticks acting as the primary vectors, and mammals and birds serving as the primary hosts. There are also orthoflaviviruses with a host-restricted range; for example, *Culex flavivirus* and *Aedes flavivirus* lack vertebrate hosts, whereas viruses like Tamana bat virus lack arthropod vectors [1,52,53]. CPCV is in the *Orthoflavivirus cacipacoreense* species [1].

*Orthoflavivirus* antigenic classification relies on serological cross-reactivity. According to the current ICTV, CPCV, Japanese encephalitis virus (JEV), Koutango virus (KOUV), Alfuy virus (ALFV), Murray Valley encephalitis virus (MVEV), St. Louis encephalitis virus (SLEV), Usutu virus (USUV), Kunjin virus (KUNV), West Nile virus (WNV), and Yaoundé virus (YAOV) share significant antigenic cross-reactivity, leading to their classification into the Japanese encephalitis virus serocomplex [1,54]. Genetic classification, based on genome sequence data and phylogenetic relationships, sheds light on the relationship among orthoflaviviruses, including CPCV. CPCV forms a paraphyletic clade with JEV, USUV, ALFV, MVEV, SLEV, USUV, KUNV, WNV, and YAOV, strongly suggesting a shared evolutionary origin with important human viruses [55].

## 3. Ecology, Vectors, and Vertebrate Hosts

Following CPCV’s discovery, studies across Brazil were focused, particularly in the 70s and 80s, on identifying its vectors and hosts of transmission [50,51]. Serologic evidence of exposure was detected in various species of birds (most undescribed species) and small- to medium-sized mammals (rodents, bats, and other undescribed species), but not universally in all study sites, revealing geographic differences in the potential role of these host species in the natural cycle of CPCV [51]. Extensive research to identify the likely vector(s) of transmission was conducted in the state of Pará, near the location where the virus was first isolated. CPCV was not isolated from *Sabethes* spp. (39 pools), *Culicidae* spp. (2252 pools), or phlebotomine sand flies (58 pools) collected in the region between 1976 and 1979. However, extensive serological testing of animals, including marsupials, non-human primates, carnivores, ungulates, edentates, and reptiles, aiming to identify the likely vertebrate hosts, was not conclusive [51].

A subsequent study performed in the state of Rondônia detected CPCV by PCR in pools of *Culex* sp. and *Anopheles* sp. [56] (Table 1). Notably, members of the *Culex* genus exhibit a global distribution range [57], are well-adapted to urban and peri-urban environments [58], and serve as vectors for several arboviruses, including West Nile virus (WNV); thus, understanding their ecology and behavior is crucial to mitigating disease transmission risks [57,58,59].

*Anopheles* mosquitoes are primarily recognized as vectors for the malaria-causing *Plasmodium* parasites [60], however, they have also been shown to be competent vectors of o’nyong nyong virus (ONNV), an arbovirus endemic in East Africa [61]. Hence, it is conceivable that *Anopheles* spp. mosquitoes could potentially transmit CPCV, although further studies are required to ascertain their vector competence. Interestingly, Figueiredo and colleagues also detected CPCV in *Ae. aegypti* pools collected in Manaus, the capital of the neighboring state of Amazonas; however, their vector competence for CPCV remains to be confirmed [56]. *Ae. aegypti*, a highly anthropophilic vector with a global distribution, thrives in urban habitats and is the main vector of transmission for many arboviruses of medical importance, including dengue, Zika, and chikungunya [62]. The spread of arboviruses by *Ae. aegypti* poses a substantial public health challenge in tropical and subtropical regions, necessitating ongoing efforts in control and prevention to minimize the impact of these diseases on the population [63,64,65].

CPCV was isolated from a pool of female ticks (*Amblyomma cajennense*) feeding on an ill capybara (*Hydrochoerus hydrochaeris*) that eventually died in the state of São Paulo, over 2000 miles away from where CPCV was originally isolated (Table 1). The isolation of CPCV from ticks could suggest that *Amblyomma cajennense* may potentially serve as a vector for CPCV transmission [55]. However, this is unlikely, since the tick females were engorged with the blood of the diseased capybara, and the detection of CPCV is attributed solely to the blood of the infected capybara. This is noteworthy, as CPCV belongs to the JEV serogroup, primarily circulating between *Culex* mosquitoes and birds [54,55].

Several surveillance studies have also been focused on horses/equines, since they inhabit peri-rural or rural environments. Rodrigues and colleagues detected CPCV seropositive horses in the states of Pará, Amapá, and Acre, all within the Amazon rainforest biome [66]. Furthermore, CPCV has also been detected in horses from other Brazilian biomes, including the Pantanal (Mato Grosso do Sul State) [67,68] and the Cerrado/Caatinga (Bahia State) [69], providing strong evidence for the local circulation of CPCV in these Brazilian biomes. Serologic detection has also been documented in water buffaloes (*Bubalus bubalis*) in the state of Pará (Amazon biome) [70] and in free-ranging non-human primates (NHP) (*Alouatta caraya*) in the state of Mato Grosso do Sul (Pantanal biome) [71]. Interestingly, caimans and sheep were also surveyed by serology, and no evidence of exposure to CPCV was reported [68].

**Figure 1:**
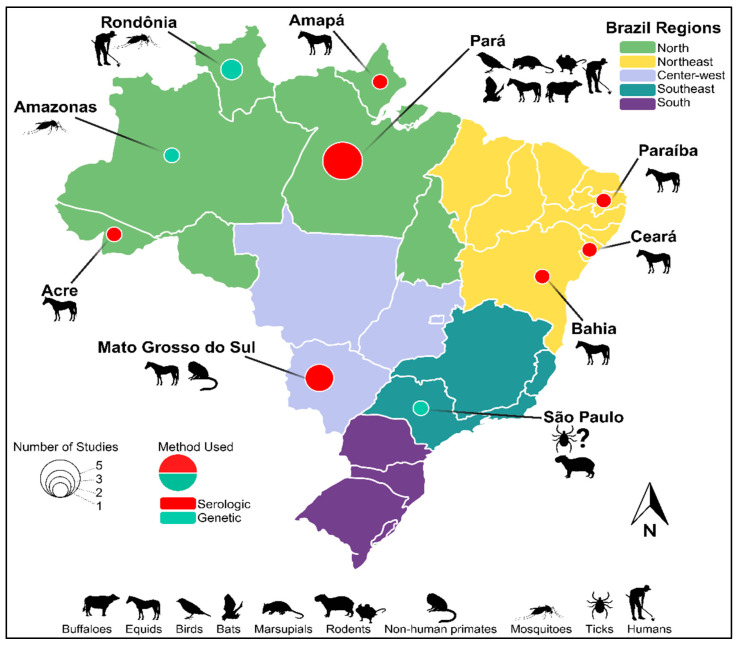
Geographic range and epidemiological landscape of Cacipacoré virus. Brazilian states with evidence of CPCV circulation are named. Hosts from which CPCV and/or antibody have been identified within a given Brazilian state are indicated by representative graphic(s). Pie charts within a given state indicate the number of studies identifying CPCV by size and the method of identification by color.

Overall, there is substantial serologic and genetic evidence of CPCV circulation, whether in wild, peri-urban, or even urban environments across various animal species or vectors in regions across Brazil (Figure 1 and Table 1).

**Figure 2:**
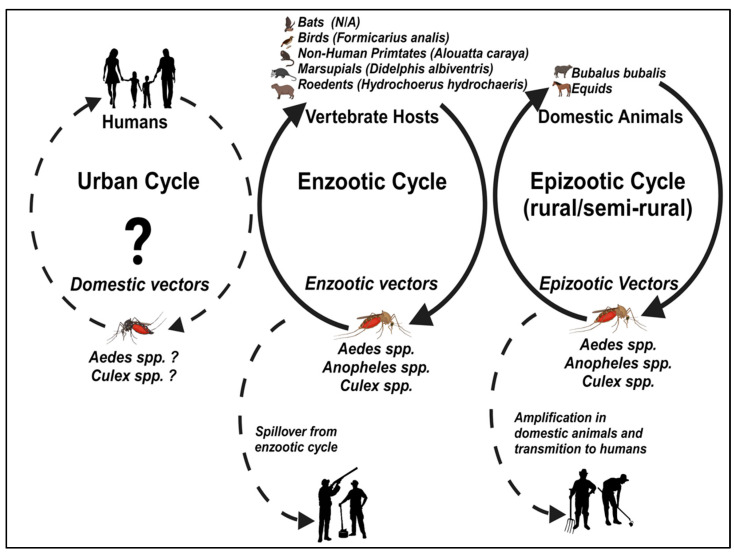
Transmission cycles of CPCV (adapted from [72]).

Regardless of the method of detection, several mammalian hosts, such as rodents, bats, horses/equines, NHPs, and water buffaloes, have been implicated in the ecology of CPCV. However, birds are believed to play a fundamental role in the natural maintenance of CPCV (Figure 2). Although the list of wild vertebrate species susceptible to CPCV infection is becoming more comprehensive, the specific role of most species in the maintenance of CPCV remains unknown. Notably, given the widespread presence of CPCV across diverse ecological settings across Brazil, it is likely that *Culex* spp. mosquitoes may play a dominant role as vectors of transmission, and birds, possibly those with migratory patterns, as amplification and reservoir hosts [56]. Moreover, the detection of CPCV in *Ae. aegypti* mosquitoes raises the potential for transmission among humans in urban settings [65].

**Table 1:** Documented circulation of CPCV among humans/animals/arthropods (adapted from International Catalogue of Arboviruses [51]).

Year	State	# of Cases	Species/Animal	Tests Performed	Refs.
1977	Pará	1	*Formicarius analis*	Viral isolation	[50,51]
1976–1979		14	Birds	HI	[51]
		1	Rodent		
1977–1980		2	Humans		
1978		2	Birds		
1979		1	Bird		
1979–1980		8	Birds		
		1	Bat		
1997	São Paulo	1	*Hydrochoerus hydrochaeris*	RT-PCR	[55]
		1 pool	Amblyomma cajennense *		
2002	Rondônia	1	Human	RT-PCR and Semi-Nested-PCR	[73,74]
2002	Rondônia	1 pool (8 females)	*Culex* sp.	RT-PCR and Semi-Nested-PCR	[56]
		1 pool (9 females)	*Anopheles* sp.		
2005–2006	Amazonas	3 pools (33 females)	*Aedes aegypti*		
2005	Pará	16	Equids	HI	[66]
	Amapá				
	Acre				
2007	Pará	1	*Didelphis albiventris*	HI	[75]
2007–2009	Paraíba	1	Equids	HI	[76]
	Ceará	10			
2009	Mato Grosso do Sul	5	Horses	ELISA	[67]
2009–2010		50	Horses	PRNT	
2009	Pará	8	*Bubalus bubalis*	HI	[70]
2009–2010	Mato Grosso do Sul	139	Equids	PRNT	[68]
2012	Mato Grosso do Sul	1	*Alouatta caraya*	HI	[71]
2013	Bahia	4	Horses	PRNT	[69]
2015	Bahia	1	Horses	PRNT	[69]
2017		2			
2018		6			

* The *Amblyomma cajennense* tick was found engorged with the blood of an ill capybara (*Hydrochoerus hydrochaeris*). Abbreviations: HI—hemagglutination inhibition test; RT-PCR—Reverse Transcriptase Polymerase Chain Reaction; ELISA—Enzyme-linked Immunosorbent Assay; and PRNT—Plaque Reduction Neutralization Test.

## 4. Human Epidemiology

Research related to the identification of CPCV in animals has been limited, and serological or molecular surveys to identify CPCV in humans are even more constrained. This further complicates our understanding CPCV’s epidemiology and pathogen–host interactions. Earlier serological surveys in humans performed in the state of Para between 1977 and 1980 showed a low prevalence in the population (2/2500 screened were positive for CPCV) and no exposure in a community of the indigenous Xicrin population (0/49) [51]. Similar outcomes were observed in two studies performed in the states of Amazonas in 1979 (0/246) and Goias in 1980 (0/622) [50]. CPCV remained forgotten until 2002, when the first isolation of the virus in humans occurred in the state of Rondônia, where a 34-year-old farmer from the city of Theobroma was admitted to the hospital with suspected YFV or leptospirosis infection. A molecular analysis of the patient’s serum confirmed a CPCV infection [73,74]. It is suspected that his occupation as a farmer may have been crucial in his exposure to CPCV. Since then, no other human cases have been detected (Table 1). Critically, caution should be exercised in the use of serologic tests in the differential diagnosis of CPCV, given the high level of antibody cross-reactivity among flaviviruses and the lack of accurate laboratory diagnostic assays, which complicate the accurate diagnosis of arboviruses, including CPCV. An additional confounding factor in the accurate diagnosis of CPCV is that most arbovirus infections present with similar symptoms and many are often misdiagnosed as dengue [49,77].

## 5. Clinical Disease, Diagnosis, and Treatment

The accurate clinical presentation of CPCV is extremely limited. To date, the only case described in the literature is the case of the farmer infected in the state of Rondônia [73,74]. According to the clinical record, there was initially a suspicion of yellow-fever-induced hepatitis. A laboratory examination revealed traces of blood in the urine, as well as reduced levels of red blood cells and hemoglobin levels, indicative of moderate anemia. Additionally, symptoms such as jaundice, hemorrhage, fever, headache, myalgia, conjunctival congestion, respiratory changes, renal insufficiency, nausea/vomiting, and diarrhea were noted [73,74]. Despite the patient being transferred to an intensive care unit, the disease outcome was fatal. Notably, during the post-mortem examination, a diagnosis of leptospirosis and CPCV infection was confirmed based on serologic and genetic tests, respectively [73]. Given that differential diagnosis was rendered during the post-mortem examination, any association between CPCV infection and the patient’s death is, at this stage, speculative. To our knowledge, to date, there are no other documented CPCV infections in humans, and thus, a precise clinical description of the disease remains unknown.

Currently, there are no commercially available diagnostic tests for CPCV except the in-house-developed serologic and genetic tests described in the literature, such as the hemagglutination inhibition test (HI) [66,70,71,75,78], ELISA, and plaque reduction neutralization test (PRNT) [67,68,69], as well as RT-PCR and Semi-Nested-PCR [55,56,79], respectively. Therefore, due to the lack of infrastructure and limited resources, a CPCV outbreak could go unnoticed and likely be misdiagnosed, given that Brazil is hyperendemic for various arboviruses and other tropical diseases presenting with similar clinical symptoms. There are also no licensed vaccines or antiviral therapies available for CPCV infections; therefore, patient care protocols include symptom management, stabilization, and intensive care unit admission for severe cases.

## 6. Prevention Options

Preventing CPCV infections can be achieved by adopting strategies common to other arboviruses. Individual protective measures against mosquitoes are crucial in averting CPCV infections and vector control is a fundamental approach, involving the elimination of mosquito breeding grounds and using screens on doors and windows [80], as well as personal protection measures, such as protective clothing, the use of insect repellents, and behavior modification to minimize human contact at peak mosquito activity [81]. Proper clothing is crucial in preventing insect bites, with recommendations for long sleeves and pants, especially during peak vector activity periods. Wearing light-colored clothing reduces exposure risks. Encouraging the use of closed shoes or boots is advised. Repellents like DEET or 0.5% permethrin for treating clothing, shoes, and equipment are recommended [82,83]. Traveling to endemic areas requires awareness of risks and taking precautions such as using insecticide-treated bed nets during sleep. Maintaining cleanliness indoors and outdoors, along with the use of repellents, contributes to a safer environment [82,83,84]. Community awareness, through educational programs and collaborative efforts towards eliminating breeding grounds, also strengthens collective defense against arboviruses [85].

## 7. Conclusions and Future Prospects

While CPCV was discovered almost 50 years ago, there is a limited understanding of its clinical presentation and disease, as well as its ecology, epidemiology, and viral genetic diversity. Serological assays for routine laboratory use in hospitals and public healthcare centers are not commercially available, hindering diagnosis in critical locations. Given the potential co-transmission of CPCV with various other human pathogens, using diagnostic panels targeting multiple mosquito-borne pathogens in an endemic area could be beneficial, providing a better understanding of disease outcomes during co-infections and guiding suitable treatment options. The development of sensitive and highly specific laboratory detection methods would be beneficial for advancing research in these areas.

The number of reported cases of CPCV infection are extremely limited, reflecting its neglected and under-researched status. Based on our review, CPCV has the potential to become an emerging threat in South America; thus, comprehensive and geographically broad epidemiological and seroprevalence studies in known biodiversity hotspots, intimately integrated with modeling approaches, may be urgently due. Epidemiological surveillance will likely identify additional potential hosts and vectors of CPCV transmission, thus informing on the potential public health risk represented by these additional vectors and hosts of transmission, which will allow us to develop effective mitigation strategies against the threat posed by emerging zoonotic and resurging arboviruses.

Despite the apparent low public health burden of CPCV infections, it should be noted that the actual impact of this virus is unknown. Information on morbidity and mortality rates and the high risk of short- or long-term sequelae in affected humans is also unknown, thus justifying the implementation of further research efforts to better understand the pathogenesis and immunity of the disease and explore new prevention and therapy options. Prevention focuses on avoiding tick and mosquito infestations through the use of individual protective measures and vector control. Vaccines against CPCV are not currently available for use, and given the low incidence of the disease, their cost-effectiveness is likely prohibitive. The known human case of CPCV demonstrated that the only care for CPCV patients is palliative. Antiviral treatment is also not available, and data on CPCV susceptibility to various antiviral drugs are nonexistent, although the growing database of antiviral drugs against flaviviruses may offer effective repurposing options against CPCV infection. Alternatively, the emerging field of antiviral treatment targeting host proteins necessary for various flavivirus cellular life cycle processes could be further explored. Unlike conventional antiviral drugs, whose routine use could easily select for drug resistance, an approach targeting the cellular components necessary for the flavivirus life cycle may have the additional advantage that resistance is less likely to develop, as host cell targets tend to evolve slowly.

Lastly, the disruption of spillover events into human agricultural habitats and emergence into urban settings will likely require novel modeling approaches that leverage a multitude of available empirical data (e.g., host range and ecotypes, etc.) that have been acquired over time by investigating similar pathogens of concern (e.g., ROCV, ZIKV, and WNV). These methods have been recently successfully employed in identifying the risk factors and drivers of zoonotic pathogen emergence [86,87] and reviewed in a previously study [5]. Note that, while history has shown us that sustainable vector control programs are the most effective methods in controlling vectors of transmission, ultimately, their success hinges on sustainable financial support by policy makers and active engagement, as well as enforcement at the community level.

## Data Availability

Not applicable.
